# Understanding Barriers to Engagement With a Prostate Cancer Research and Genetic Risk Service Among UK Men of Black African or Black Caribbean Ancestry

**DOI:** 10.1111/hex.70282

**Published:** 2025-04-29

**Authors:** Hall Rose, Emma Hainsworth, Jeff Thompson, Saran Green, McGrowder Eva, James Denzil, Eeles Rosalind, Elizabeth Bancroft

**Affiliations:** ^1^ The Institute of Cancer Research London UK; ^2^ The Royal Marsden NHS Foundation Trust London UK; ^3^ Cancer Don't Let it Win London UK; ^4^ The Patient and Public Involvement Cancer Research Group for Diverse Backgrounds London UK

**Keywords:** Black African and Black Caribbean ancestry, cancer screening, genetic testing, health equity, prostate cancer, representation

## Abstract

**Introduction:**

Prostate cancer is the second most common cancer worldwide, and there is no national prostate cancer screening programme in the United Kingdom. Men of African ancestry are twice as likely to be diagnosed as men of European ancestry and are diagnosed at a younger age. Despite this, Black men are under‐represented in seeking advice about prostate cancer symptoms, screening and genetic research. There is increasing research focused on targeted prostate cancer screening, using genetic testing to guide screening by identifying those at highest risk, but this could only be considered if people of all ethnicities would accept this approach. It is vital to diagnose prostate cancer early, when it is curable. We wanted to identify the barriers to engagement with prostate cancer genetic research to increase participation from those at highest risk.

**Methods:**

We conducted two community discussion groups, each attended by 30–35 Black men and their families. We conducted interviews with three Black community champions who have a lived experience of prostate cancer. Thematic analysis was performed on the transcripts. We used a participatory approach to develop our themes with members of the community, two of whom are co‐authors on this paper.

**Results:**

Themes were grouped as barriers or facilitators to engagement with prostate cancer genetic risk services. Barriers included GP reluctance to perform prostate‐specific antigen (PSA) testing, cultural inhibition around discussing prostate cancer and family history, fear of rectal examination, fear of cancer diagnosis and lack of trust in the healthcare system, no awareness about the role of genetics in prostate cancer risk assessment, negative connotations of genetic testing (e.g., genetic modification) and genetic data being used inappropriately. Facilitators were family and community support, the sharing of experiences, good communication with doctors, raised prostate cancer awareness, genetic risk assessment to guide the need for screening and facilitate early diagnosis, improving future outcomes for prostate cancer in the Black community through engaging with genetic research and assurance that there are regulations in place to protect genetic and personal data with guidance around when genetic results must be disclosed.

**Conclusions:**

Understanding barriers and facilitators can guide recommendations for health services to improve access and uptake within the Black community and improve representation in genetic research. Better representation will support improvements in cancer outcomes and understanding of the genetic risk of prostate cancer in the Black community.

**Patient or Public Contribution:**

We initially attended community prostate cancer awareness events to speak to members of the community. We established trusted and two‐way relationships with Black ‘community champions’ who lead support groups in the Black community and often have a lived experience of prostate cancer. We were invited to attend their support groups to deliver awareness talks and address concerns about prostate cancer risk and screening. We then conducted discussion groups and collected data. Our analysis was conducted in partnership with our community champions. Our findings are described in this paper, with their co‐authorship. We have also disseminated our findings in a co‐produced newsletter to feed back our findings to the community members, who gave us their time. We have also shared information at a stakeholder day, attended by 65 individuals from the community, where we also planned future work. We have reimbursed participants for their time, which is in line with NIHR guidance. As described above, patient and public involvement has been the guiding principle throughout this project.

## 1 Introduction

This paper reports a patient and public involvement project conducted in partnership with men of Black African and Black Caribbean descent. Our genetic risk assessment service provides genetic testing and prostate cancer screening within a research setting to individuals who are at elevated risk of prostate cancer, including those with Black African and Black Caribbean ancestry. We have low representation in participants from this demographic, despite nationwide recruitment through GPs and outreach initiatives. Therefore, we wanted to find out why Black men were under‐represented in our research service. Our aim was to better understand Black men's views and perceptions about prostate cancer, genetics and screening and to identify any potential barriers to engagement with a prostate cancer genetic risk research service. This knowledge is important to inform the design and delivery of our health services, information materials and research, to make them more accessible and inclusive to this underserved group.

Prostate cancer is the most common cancer in men and people born with male reproductive organs in the United Kingdom. It can be slow growing and relatively harmless (indolent), or it can spread around the body to be life‐limiting (advanced or aggressive) [1]. In the United Kingdom, approximately 55,000 men are diagnosed with prostate cancer every year (144 per day), and there are 490,000 men living with/having had the disease [2], and incidence is rising [2]. Detecting prostate cancer in the early stages is important because it is curable when retained inside the prostate at diagnosis (i.e., has not spread to other areas in the body). Later diagnosis, when the cancer has spread outside of the prostate, leads to worse outcomes, with only half of men surviving for 5 years.

The ambition of the NHS long‐term cancer plan is that by 2028, 75% of people with cancer will be diagnosed at an early stage (Stage 1 or 2). There is no established national screening programme for prostate cancer in the United Kingdom, and the existing method of testing in NHS primary care is a blood test for prostate‐specific antigen (PSA) [1]. PSA testing is offered to men who have symptoms suggestive of prostate cancer or who request a test, after a discussion with their GP about the pros and cons of using it as a risk assessment tool.

In the United Kingdom and the United States, a prostate cancer risk discussion in primary care usually involves a medical and family history, a digital rectal exam and a serum PSA test [3, 4, 5]. If these indicate the possibility of prostate disease, imaging such as an MRI scan will guide who should receive a prostate biopsy [6]. Using serum PSA for prostate cancer screening in asymptomatic individuals is a controversial topic with no international consensus [7]. In advanced prostate cancer (high Gleason scores), PSA sensitivity is 80%–90% [8]. There are large datasets which report that PSA screening in the general population reduces mortality by approximately 20% [9]. However, screening with a PSA test alone cannot distinguish between clinically significant or indolent prostate cancer, which can result in unnecessary prostate biopsies, overtreatment (with potentially life‐changing side effects of incontinence and erectile dysfunction) and psychological burden [10]. Some argue these harms outweigh the benefit of mortality reduction. To reduce the overdiagnosis of indolent prostate cancer, there is research focused on targeting PSA testing to men who are at increased risk of the disease. Prostate cancer is the most heritable cancer, and research is evaluating whether genetic testing can be used to estimate an individual's lifetime risk of developing the disease and therefore guide which individuals would benefit from a future prostate cancer screening programme.

Established risk factors for prostate cancer include age, ethnicity and having a family history of the disease [10]. Ancestry is a known risk factor for prostate cancer, and those of Black African and Black Caribbean ancestry (whom we will refer to as ‘Black men’) have a higher risk than those of White European ancestry (whom we will refer to as ‘White men’). The risk for Black men is 1 in 4, compared with 1 in 8 in White men by age 80. Black men are also more likely to be diagnosed at a younger age. A 2023 study showed that Black men have the same prostate cancer risk at age 66 that White men have at age 85 [11]. Therefore, Prostate Cancer UK (PCUK) recommend that all Black men should be offered a PSA test if they request one from age 45 and above [3, 12].

Until recently, there has been little genetic research in Black African and Black Caribbean populations, which meant we did not know for certain whether genetic variation could explain this higher rate of diagnosis (incidence) and death from prostate cancer (mortality). This knowledge gap is shrinking, and our understanding of the genetic contribution to the risk of prostate cancer is improving [11]. In 2009, 96% of participants in Genome Wide Association Studies (GWAS) were of European ancestry, with less than 1% accounting for those of African ancestry [13]. By 2016, the representation of those with African ancestry had risen to 3% [14]. This small proportion is not representative of the Black population worldwide; however, there are limited data stating the percentage of the world population that is Black. In the United Kingdom, 4% of the population of England and Wales has Black African or Black Caribbean ancestry, and within London, where our clinic is based, this is 13.5% [15].

Genetic variants have been identified that increase the risk of prostate cancer, and some of these have a different incidence in Black men compared with other ethnic groups and may provide some of the answer as to why Black men are more likely to get the disease and be diagnosed at a younger age than White men [4, 5]. We have ongoing research studies that are using genetic testing to identify men at higher risk of prostate cancer for targeted prostate cancer screening. Black men are under‐represented in this study. It is important to encourage more Black men to take part in prostate cancer genetic research so that we can continue to collect data, which can improve our understanding of risk and promote better prostate cancer outcomes for this community in the future.

Whilst some of the risk is thought to be due to genetic differences, the impact of health behaviours and interactions with health services also contribute to how and when people are diagnosed with prostate cancer, and health inequalities exacerbate this problem. Much of the existing literature describing the barriers that Black men face when accessing a prostate cancer risk discussion (which includes a medical and family history, digital rectal exam and serum PSA test) comes from the United States. Healthcare accessibility and health insurance coverage can impact this group's access to screening [7, 8, 9]. The literature also describes a lack of awareness of prostate cancer risk amongst Black men and a reluctance to engage with healthcare services in the absence of a health problem [10]. Studies also reported a lack of conversation within the community about health and cancer [9, 11].

Black people with a significant family history of cancer are less likely to access clinical genetics services. Barriers could include cultural inhibitions around discussing family history, the need to complete a complex family history questionnaire [16, 17], discussing how relatives died or whether it was disclosed that a relative had cancer, with some expressing views that cancer is a ‘new condition’ and an issue only affecting White people [11].

Finally, trust in healthcare systems has been highlighted in the literature as a barrier to Black men accessing advice about prostate cancer. Studies have reported examples of poor communication [10, 14], lack of GP knowledge about increased prostate cancer risk among Black men and instances of inconsistent information being given regarding PSA testing.

To summarise, our study is the first to our knowledge to describe attitudes within the Black community to the incorporation of genetic risk assessment in cancer risk prediction and screening. There is a lack of evidence from the United Kingdom describing the barriers and facilitators faced by Black people when seeking advice about prostate cancer. There is also a lack of representation of Black people in cancer genetic services and genetic databases [17]. Our particular focus is on the United Kingdom and on understanding views about using genetic testing within a prostate cancer risk assessment, and we are building on previous work in this space to establish trusted relationships between our genetic risk service and Black communities around London and further afield. We hope these partnerships will educate us about the needs of Black people in accessing cancer screening and advice. In return, we hope to develop the trust of communities and share information around personal health, cancer risk and accessibility to screening and risk advice.

The aims of this co‐design work were to work in partnership with men of Black African or Black Caribbean descent to better understand barriers to engagement with the prostate cancer genetic risk research service and explore information requirements and ways of working, which could increase their participation in prostate cancer and genetic research.

## 2 Methods

### 2.1 Data Collection—Discussion Groups and Interviews

We attended fundraising, awareness and social events to become familiar with the prostate cancer awareness groups within the Black community, and as a result of this, we met the community champions who invited us to their support groups, where we held our discussion groups. These two discussion groups were facilitated by the community champions (one of whom is a co‐author on this paper) and were attended by Black men and their families. They occurred in community centres in two separate Boroughs in South London. By visiting the men in their own environment, surrounded by friends and family, we hoped to empower them to share their opinions and lived experience.

The composition of these groups was as follows:

- Each was attended by between 30 and 35 men and women (a ratio of approximately 70% men and 30% women) from the Black community.
- The majority of attendees had a lived experience of prostate cancer.
- Attendees were multigenerational, and ages ranged from 18 years old and above, though the majority were aged 50 and above.
- Both focus groups were distinct, with separate organisers and attendees.
- Most attendees were of Black Caribbean ancestry, rather than Black African ancestry (this is anecdotal information from the organisers of the events).
- We did not keep any personal and demographic data regarding the individuals who attended, as we were concerned that this could be a barrier and deemed it unnecessary.

In the spirit of reciprocity, we provided a presentation at each group, followed by a question‐and‐answer session to raise awareness about prostate cancer and allow attendees to ask questions that were of concern to them. After the presentation, we used a topic guide consisting of a few open questions, which were put to the group to prompt discussion about their views and understanding of genetics, research and prostate cancer risk more generally. They were asked to think about potential barriers to engagement and consider what kind of messages might help Black men overcome any of these barriers (see topic guide Additional File 1). Both our presentation and topic‐guide questions were reviewed, approved and finalised by the community champion who organised the first session and is also a co‐author of this paper.

Members of the group bounced off one another, endorsing or disagreeing with statements made. The conversation was always flowing, and the whole group were united in one conversation at one time, and we steered the discussion back on track if it digressed. We gained approximately 2.5 h of audio per discussion, and there was significant overlap and recurrent themes in both groups. We did not note any contrasting themes from the groups. We made a donation to the organisers of the community groups, and we continue to nurture long‐term and two‐way relationships with them.

We conducted one‐to‐one interviews with three Black community champions via video call. With interviewee consent, we gained an hour of audio from each interview, and the themes echo those from the discussion groups. One of these community champions is the founder of the organisation that accommodated our first discussion group, and the other two we met as attendees to our second discussion group. All three community champions were renumerated for their time, and we continue to foster long‐term and two‐way relationships with them. Two of the community champions are female, and they both have a family history of prostate cancer in their close relatives. All three of these champions either run or assist in the organisation of prostate cancer awareness events in the Black community.

This project was approved as a service evaluation (CCR SE1284). With verbal consent, we audio‐recorded 3 h of discussion in total from the two groups.

### 2.2 Guided by Principles of Co‐Production

For this study we use co‐production to describe an approach based around a set of key principles. Those principles are power‐sharing, building trusted relationships, reciprocity and shared learning [18, 19, 20]. Rather than a top‐down knowledge ‘translation’ [21], this approach uses the generation of knowledge through collaboration between researchers. It goes beyond patient involvement collaboration models, which can result in imbalances of power with researcher‐led agendas [22, 23]. We wanted to conduct our involvement activity using a more equal power relationship between experts by experience and researchers, so that the output was determined by the communities with which we worked in partnership.

Two of the community champion interviewees have co‐authored this paper. One is the founder of the organisation that facilitates help, support, advice and awareness events for prostate cancer in the Black community, and he himself is a prostate cancer survivor. We shared the extracted themes with him for his assessment. This paper was planned and drafted with him and our other community champion co‐author, and they also revised the drafts to agree on the final version. They have written paragraphs in ‘Results’ and ‘Discussion’ sections, and they have edited all sections of the paper.

### 2.3 Data Analysis

Data from the discussion groups and interviews were digitally recorded and transcribed verbatim. The transcriptions were thematically analysed manually; the process was aided by reflexive notes [24]. This approach involved two members of the project team (E.H. and R.H.) generating initial codes from the transcripts, then reaching coding consensus, agreeing on categories and inductively developing broader themes. To ensure the reliability of the final themes, these were checked against the participants' initial responses, and the data were peer reviewed by other members of the project team and with our public co‐authors [25].

### 2.4 Writing Up the Involvement Project for Publication

We wanted the collaborative nature of this project to extend beyond the interviews and discussion groups and into the future information‐sharing stages. Two of the community champions agreed to co‐author this paper. We also consulted the Race, Ethnicity and Cultural Heritage (REACH) staff forum for the Royal Marsden Hospital and The Institute of Cancer Research to review and approve the language used in this paper.

## 3 Results

Thematic analysis of the data identified the following recurrent key themes summarised in Table 1 and further described in the narrative description below.

**Table 1:** Table of key themes with quotes extracted from transcription data from focus groups and interviews.

1. Category—Interactions with the GP—perspectives and experiences	
Sub‐theme	*‘If it ain't broke why fix it?’* hard to persuade men to go to GP when they feel well, with no symptoms
	*‘If I haven't got any symptoms, why bother going to the GP?’*
Sub‐theme	Hard to get appointments with GPs
Sub‐theme	*‘the first thing the GP says is no’* perception that a request for a test will be met with resistance
	*‘the common story, when you go to the GP, the first thing the GP says is no. So in my case, my dad died of it, he* (the GP) *knew about it, but he still said no’* *‘there are many who have been turned away at their GP office because they didn't qualify for whatever reason’*
Sub‐theme	Confusion about what age you can request a PSA blood test from the GP (current guidance is that Black men should receive PSA testing from age 45).
	*‘you should be informing families who have kids and letting them know about 30 years they should be having a PSA’* *We've got younger men in our families now, we need to encourage them to have themselves checked out as early as possible’* *Suresh ‘You're entitled to tests from the age 50 under the prostate risk management programme. However, we tell black men and men with family history to go at age 45, whether you have symptoms or not’*
Sub‐theme	The taboo associated with the digital rectal examination can be a powerful deterrent
	*‘the people I speak to they will not allow the doctor to touch them. It's a black man thing, being touched by another male, they just don't want it’* *‘there's thousands of guys out there who think the prostate test is a finger up the rectum and don't know it's just a simple blood test’* *‘I tell people to think of it like a cholesterol test. I point to my arm to show that it is a normal blood test, forget about the finger’* From a man who has been through treatment for prostate cancer *‘for those talking about DRE, from someone who has had other examinations I would say to you let's be real… I can assure you there are worse things … that make a DRE seem like child's play’*

2. Category—Some cultural inhibitions talking about prostate cancer	
Sub‐theme	Some families don't share information about who has had cancer—particularly older generations—this has implications for assessing family risk
	*‘my family was actually furious with me for digging into our foundations when I didn't have any problems’* *‘I think there's an education thing in our community, there are a lot of people that don't know their family history or how people have died … their parent passes away and they don't know this is what they had’* *‘my father didn't tell me anything about it and I was wheeling him about all over the place to visit urologists…I didn't know until he was really sick that he had prostate cancer’*
Sub‐theme	Fear of discussing a sensitive topic such as prostate cancer & links to masculinity
	*‘I think culturally as black men, we don't openly discuss prostate cancer or if we're having trouble downstairs or whatever and also I think there's a lot of fear’* *‘Because it's their manhood isn't it? It's something beyond their control, if everyone were to know I had prostate cancer, I can't have sex. So therefore ‘zip’ I'm not going to talk about it’*
Sub‐theme	Not wanting to know—Denial, being silent
	*‘They don't want to know, they don't want to hear it's a problem’* (one man talking about younger cousins in a large extended family) *‘I went to an event where a man said if I don't think about it, it won't come my way.’* *‘Our black men and our family and friends need to be more vocal and visible. It is not a private matter. It is not something we should be afraid to discuss in our families, in our social groups in our churches…the fact is we are too silent, we like to be seen as good men and women’*

3. Category—Lack of trust in research (and NHS) generally	
Sub‐theme	Widespread awareness within the community of historical cases where black people have been mistreated in research
	*‘That's what's stopping us. And Henrietta Lacks. Her cells were used without permission, so they don't trust. If you say you are doing research, they don't trust you. They don't know what you are going to do with that data. Are we going to get anything back from it’* *‘Plus there is a history, often people don't want to talk about the history, of black people being used as guinea pigs. There's the Tuskegee research project and there are other cases in the memories of our people that are passed on over the years by word of mouth and then the Covid and all the information that went about with Covid—people are distrustful’*
Sub‐theme	Perception black people not treated as well within NHS and worry about the use of data
	*‘Just as for pregnant women and birthing mothers “we are not at the top of the agenda.”’* *All the things that are happening in data, maybe having some more information about what you do with data, about how it doesn't need to be shared, things like that, just to put people's minds at rest. The data is not going to be given to anyone else*

4. Category—Thoughts about genetics and prostate genetic screening	
Sub‐theme	Associations with the term ‘genetics’ (what do you think of when you hear the term ‘genetics’?)
	*‘Family’ ‘Family history’ ‘Bloodlines’ ‘Ancestry’ ‘Sickle cell’* Less positive associations *‘genetic modification’ ‘cows with two heads’ ‘GM’ ‘bad Frankenstein foods’*
Sub‐theme	Thoughts about genetic testing for prostate cancer—unaware of it—positive about it in principle—concerns about insurance implications
	*‘I'd never heard of it before. My father, brother had prostate cancer, my mother breast cancer, two uncles prostate cancer. I'm probably the only one of two alive in my family that doesn't have cancer, so this would be something…’* *‘Anything I can do to help either minimise or get rid of this awful disease … as you can see I've got a little man (son), anything I can do to stick around and help him’* *‘It won't be too long before insurance companies begin to ask about genetic testing. If you say you have been tested, then that opens the door for them to say ok what were the results? And if you don't want to share it, they will think there must be something there’*

5. Category—How can we communicate better about prostate genetic screening?	
Sub‐theme	Messaging and information are more impactful if they come from lived experience
	*‘You need a live experience, it's alive, it's not made up. It's not something like reading in a book, I've lived this’*
Sub‐theme	Go to (or take the messages) to where black men, their family and friends are
	*‘You need to go to the black man but also his family and friends. Barber shops, clubs and churches, where they congregate’* *‘I don't know how easy it is for you to do genetic collection not in a hospital location—so it might be something to think about in terms of how you partner with groups and come into the community, for example go to a church or mosque, give them the information and maybe give them the opportunity to do it* (participate in genetic testing) *right there and then’*
Sub‐theme	Sell the message that there is a lack of data from the black community, which potentially affects their outcomes
	*‘this is the information we need to be sharing, you need to highlight there's this lack of data, lack of information in our community and the impact that that is having on outcomes for patients from our community’*
Sub‐theme	Importance of reassurance about research governance, and particularly the protection of data
	*‘All the things that are happening in data, maybe having some more information about what you do with data, about how it doesn't need to be shared, things like that, just to put people's minds at rest. The data is not going to be given to anyone else’*

### 3.1 GP Interactions

There were a number of issues highlighted that impacted men's relationships with their GP and seeking advice about prostate cancer. A common theme was the reported difficulty in getting a GP appointment. Men also talked about their tendency to only seek medical advice to address an actual problem rather than thinking about detecting a future one through screening.

Some of the participants described their experiences of being refused PSA tests by the GP or encountering confusion about the appropriate age to request them. This could then act as a deterrent to seeking any further prostate cancer risk advice and could create potential mistrust in their relationship with their doctor.

‘There are many who have been turned away at their GP office because they didn't qualify for whatever reason’

A frequently raised theme was the taboo and cultural stigma around the digital rectal exam, and the impact of this on men's decision to avoid seeking screening or advice about prostate symptoms. This taboo, whilst an issue for many heterosexual men, was particularly vocally expressed by the men we spoke to:

‘The people I speak to, they will not allow the doctor to touch them. It's a Black man thing, being touched by another male, they just don't want it’

### 3.2 Cultural Inhibitions Around Discussing Prostate Cancer

We learned from several participants that some families in the Black community do not talk about how relatives died and that members of the older generation, particularly, may not share their cancer diagnoses with their family. This makes it difficult for Black men to assess their own individual prostate cancer risk if they do not know their family history.

‘My father didn't tell me anything about it and I was wheeling him about all over the place to visit urologists…. I didn't know until he was really sick that he had prostate cancer’

‘There are a lot of people that don't know their family history or how people have died … their parent passes away and they don't know [prostate cancer] is what they had’

The men reported a widespread fear of discussing prostate cancer because it is a sensitive topic, which links to a man's sense of masculinity and his perceived ability to perform sexually.

‘Because it's their manhood isn't it? It's something beyond their control, if everyone were to know I had prostate cancer, I can't have sex. So therefore “zip” I'm not going to talk about it’

Men also described a prevailing belief amongst their peers that in the very act of thinking about the prospect of ill health, they may somehow bring it into being.

‘I went to an event where a man said if I don't think about it, it won't come my way.’

There was a consensus in the group that if men felt enabled and empowered to talk about their health and cancer, it would make it feel normal and encourage others to do the same, which would be a good thing.

### 3.3 Lack of Trust in the NHS and Research

There is widespread awareness within the community of historical cases where Black people have been mistreated and exploited within research. Examples of these cases were described by members of the group, providing a rationale for some people's current lack of trust in research.

‘That's what's stopping us. And Henrietta Lacks. Her cells were used without permission, so they don't trust. If you say you are doing research, they don't trust you.’

As well as historical examples of injustice within research, such as the Tuskegee syphilis project and Henrietta Lacks in the United States, lack of trust in the NHS was also described more generally and evidenced more recently with misinformation in the community around Covid‐19 and the poorer maternal outcomes of Black women within the NHS.

‘Plus there is a history, often people don't want to talk about the history, of Black people being used as guinea pigs. There's the Tuskegee research project and there are other cases in the memories of our people that are passed on over the years by word of mouth and then the Covid and all the information that went about with Covid—people are distrustful’

This mistrust could coalesce around the use of data by researchers and the NHS more generally. There was consensus expressed in the groups that more work could be done to inform and reassure people in the Black community about the appropriate handling of data.

‘All the things that are happening in data, maybe having some more information about what you do with data, about how it doesn't need to be shared, things like that, just to put people's minds at rest.’

To rebuild the community's trust in medical research, transparency is crucial. Providing detailed information around the aims and purpose of studies, as well as sharing how researchers ensure samples and data are protected, is important.

### 3.4 Thoughts About Genetics and Prostate Genetic Screening

In response to our questions around what the term ‘genetics’ makes you think of, we found a mixed response of some generic thoughts such as (‘Family history’ and ‘Ancestry’) and some with negative associations (‘genetic modification’ and ‘GM food’).

We explored thoughts about genetic testing for prostate cancer, and some reported that they were not aware of it.

‘I'd never heard of it before. My father, brother had prostate cancer, my mother breast cancer, two uncles prostate cancer. I'm probably the only one of two alive in my family that doesn't have cancer, so this would be something…’

Many were positive about it in principle and saw the benefits for their own children and future generations.

‘Anything I can do to help either minimise or get rid of this awful disease … as you can see I've got a little man (son), anything I can do to stick around and help him’

A few men expressed concerns about any potential insurance implications.

‘It won't be too long before insurance companies begin to ask about genetic testing. If you say you have been tested, then that opens the door for them to say ok what were the results? And if you don't want to share it, they will think there must be something there’.

This suggests the Black community are positive about participating in genetic research for prostate cancer, provided they are given information about the aims and purpose of the study and how their data are used and protected. The importance of counselling alongside genetic results to clarify their meaning in terms of health prospects for them and their families would be essential, with long‐term access to support and advice.

The participants also want clear guidance around insurance implications and how a genetic test result that identifies a pathogenic variant will affect the ability to get health, travel and life insurance. Some individuals were concerned that carrying a pathogenic variant in a cancer gene may affect their ability to get a mortgage. As of early 2025, predictive genetic test results through research do not need to be declared to third parties. According to the Code on Genetic Testing and Insurance, which is a long‐standing voluntary agreement between the UK government and Association of British Insurers, insurers will only consider the result of a predictive genetic test for a very small minority of cases (to date, only that for Huntington's disease in life insurance applications for a financial limit greater than a total of £500,000) [26]. However, it is possible that this will change in the future.

There was also concern around safe storage of genetic data and potential implications should these data be stolen and used for unregulated or unintended purposes. Those who expressed these concerns referenced the Henrietta Lacks case as an example of human tissue being used for purposes out of the scope of the patient's consent.

### 3.5 Better Communication Around Prostate Genetic Risk

We asked the participants for their advice on how we can improve our research service and make it more accessible and acceptable to the Black community. Several participants stressed the importance of being proactive and taking the information, and if possible, the research, out to the community.

‘You need to go to the Black man but also his family and friends. Barber shops, clubs and churches, where they congregate’

Many were in favour of messaging that involved the sharing of real people's stories, as this was thought to be more impactful:

‘You need a lived experience, it's alive, it's not made up. It's not something like reading in a book, I've lived this’

It is important to the Black community that their health outcomes are improved now and for future generations; therefore, it should be emphasised that there is a lack of genetic data from the Black community. The individuals we worked with felt that if this was communicated to them and circulated within the community, it would incentivise people to participate in research to provide greater representation of those with Black African or Black Caribbean ancestry.

The above themes demonstrate the thoughts and priorities around communicating about prostate cancer risk and genetic screening in the Black communities with which we collaborated.

## 4 Discussion

Our findings around the barriers faced by the Black community in accessing advice about prostate cancer risk align with many of those described within the wider literature. We will focus our discussion on views and understandings relating to prostate cancer and genetics.

Historical and generational trauma based on past and present health and research injustices has resulted in the Black community having a general mistrust in healthcare and research, which spans the United Kingdom and the United States. Scandals in the United States, such as the Tuskegee Syphilis study and the Henrietta Lacks case, detail the exploitation of Black people by medical research within the last 100 years, creating the belief that in recent history Black people have been used as ‘guinea‐pigs’ by scientists [27]. We were also told about recent experiences of miscommunication between Black men and their GPs around prostate cancer risk and PSA‐testing eligibility, which may exacerbate pre‐existing mistrust. This is a challenging area as there is no clear national guidance for GPs to follow, and the recommendation is for shared decision‐making about the appropriateness of PSA testing, including discussion around the pros and cons of screening.

Our findings agree with studies that describe cultural beliefs among the Black community in the United Kingdom and the United States around prostate cancer management being a threat to masculinity [16, 28, 29, 30], with a stigma that this diagnosis affects virility and sexual function [27, 29, 30, 31]. There is a belief that undergoing a rectal examination is a traumatic and humiliating invasion of privacy, with some believing it might cause a change in sexual orientation [27, 29, 30, 31]. This stigma deters men from sharing their diagnosis with their families and community.

We also echo the evidence that the fear of receiving a cancer diagnosis is enough to deter some men from seeking screening [29, 30, 31]. There is a fear of seeing a doctor [27, 30]. It was discussed that there are ideas around a cancer diagnosis being equivalent to a death sentence [30, 31], with a gap in understanding around treatability and even curability if diagnosed in early stages. Studies describe feelings of shame and embarrassment associated with receiving a diagnosis of prostate cancer [27, 29]. Both a perceived threat to masculinity and fear of a cancer diagnosis may be barriers to men seeking genetic screening to assess their own risk of prostate cancer.

This paper is the first to describe the barriers and facilitators faced by men in the Black community when accessing genetic testing as part of research into prostate cancer risk. Most of the men who worked with us had no awareness that this was possible, and they were open to the idea of anything that optimised their opportunity of catching the disease early and reducing harm from the disease for their generation and future generations. This would require long‐term support for those who receive a genetic test result showing that they carry a cancer‐predisposition variant. First, we need to provide accessible and acceptable educational materials to describe what is involved in the genetic research, the benefits to the individual and why it is important to increase representation of those with Black African and Black Caribbean ancestry in genetic research. The materials should also explain what data protection regulations are in place to protect their genetic data, with reference to insurance and mortgage implications. We are using the barriers and facilitators described above to co‐design and co‐produce these materials with the community champions who continue to work with us.

Despite these barriers, this study has highlighted ways to ensure that future collaborative work has the potential to improve communication about prostate cancer risk. Community champions sharing their story with their communities normalise talking about cancer and can address myths and fears around cancer screening [27]. This is especially effective when young people in the community witness this behaviour as culturally accepted among seniors [29]. An African American study stated Black men are more likely to trust health advice from their community than from healthcare professionals [27].

The NHS is working with these champions to find ways to make their services more accessible. Face‐to‐face communication with trusted healthcare professionals to discuss risk and prevention promotes engagement [17]. An effective example of this is the Newham Prostate Health Drop‐in clinic in London, which is based at Newham African‐Caribbean Resource Centre [32]. The aim of the clinic is to be accessible to men who feel excluded by the healthcare system and is facilitated by a self‐referral service and flexible opening hours [32]. Those who attended the clinic felt satisfied with the service, which made a number of new diagnoses of early prostate cancer [32].

It is described that Black men are motivated to see the doctor by encouragement from family members (especially spouses) and the need to be well to care and provide for their family [30, 31]. Support from families and communities is essential to the prostate cancer treatment journey and recovery, with churches also being highly valued [28, 29, 30]. It is widely accepted that the most effective way to raise awareness about prostate cancer and research in the Black community is through sustained community‐based projects [33], of which there are now many in the United Kingdom (see Prostate Cancer UK online database for a full list of these [34]). These provide peer support for those going through cancer and spread awareness with signposting to screening services.

Two of the community champion interviewees from our project form a public patient involvement (PPI) panel who are co‐producing educational web materials with us. They have planned what information should be included in the materials, and once complete, these materials will be disseminated by them within their community networks, to raise awareness of prostate cancer risk and genetic risk.

Outcomes for prostate cancer in Black men can be improved with better communication from healthcare professionals and community initiatives to raise awareness about prostate cancer risk and symptoms. Our findings suggest that people from the Black community are open to participating in research and genetic testing for prostate cancer, provided they are given information detailing how their data are used and protected. They also want clear guidance around declaring genetic risk to insurers and mortgage companies. It is important to many of the people that we worked with that health outcomes for future Black generations are better and equitable, and we believe that open communication around risk factors, including genetics, will help to achieve this.

### 4.1 Limitations

Limitations of this study include our discussion groups occurring only in London. We attended community groups within two separate Boroughs in South London to collect the data. We have since engaged with community groups across the country, where we continue to deliver awareness talks and are building collaborative relationships. The discussion group attendees were a large sample size (a total of approximately 60 Black men), with a high majority of these participants sharing their ideas in group discussion. The large majority were of Black Caribbean ancestry; however, we did not assess ethnic sub‐groups or collect other demographic data. It is possible that these 60 Black men from around London do not represent the breadth of diversity in lived experience in the Black communities across the United Kingdom.

The two interviewers have no Black African or Black Caribbean ancestry themselves, and we are undertaking this study with the intention to improve prostate cancer awareness, screening and outcomes in this population. We acknowledge that it would be ideal if those who conduct this study were of Black ethnicity. Consequently, we have worked hard to prove long‐term commitment to engagement with these community groups and to listen to them, to develop a trusting and two‐way relationship. We have worked closely with the community champions to guide us in making culturally appropriate use of language and ideas and show us how best to serve their community.

The knowledge that we have gained from this study will change our practice forever, by educating us around the history of Black people in medicine and research and awarding us a cultural understanding of the barriers and facilitators faced by the community when accessing healthcare services. We aim to use this understanding to break down these barriers in our clinical work, and we have delivered presentations to our colleagues to share ways individuals can change their practice to make a positive difference to accessibility for the Black community at our institution.

## 5 Conclusion

This is the first example of work describing attitudes towards genetic testing as part of future cancer screening within the Black community. It includes learning about what information should be provided to promote engagement. Our findings have supported those from previous studies in the United Kingdom and the United States regarding barriers to Black men accessing prostate cancer risk advice, but also provide new information about their perspectives on genetics in risk measurement.

We identified trust in the healthcare system, prostate cancer risk awareness and cultural inhibitions around talking about prostate cancer and family history as barriers, as well as fear and taboo around the rectal examination. Facilitators included community‐based initiatives to raise awareness of risk and sharing the lived experience of prostate cancer from within the Black community.

People from the Black communities we engaged with are open to the future incorporation of genetic testing to guide who should receive prostate cancer screening, provided sufficient information around data use and protection and insurance implications are stipulated. There is recognition of the need to improve prostate cancer outcomes within their community, and they welcome community engagement by healthcare services to facilitate this.

## Author Contributions

**Hall Rose:** investigation, writing – original draft, methodology, writing – review and editing. **Emma Hainsworth:** methodology, validation, writing – review and editing, investigation. **Jeff Thompson:** writing – review and editing, conceptualisation, resources. **Saran Green:** conceptualisation, writing – review and editing, resources. **McGrowder Eva:** conceptualisation, investigation, validation. **James Denzil:** conceptualisation, investigation, validation. **Eeles Rosalind:** supervision, writing – review and editing, visualisation. **Elizabeth Bancroft:** visualisation, writing – review and editing, supervision.

## Ethics Statement

This project was approved as a service evaluation, therefore there was no ethical approval required.

## Conflicts of Interest

Professor Rosalind Eeles has the following conflicts of interest to declare: Honoraria from GU‐ASCO, Janssen, University of Chicago and Dana Farber Cancer Institute USA as a speaker. Educational honorarium from Bayer and Ipsen, member of an external expert committee to AstraZeneca UK and member of the Active Surveillance Movember Committee. She is a member of the SAB of Our Future Health. She undertakes private practice as a sole trader at the Royal Marsden NHS Foundation Trust and 90 Sloane Street SW1X 9PQ and 280 Kings Road SW3 4NX, London, the United Kingdom. No other authors have a COI to declare.

## Supporting information

Additional file 1 Topic Guide.

## Data Availability

Data supporting this study cannot be made available as participants did not agree for the transcripts to be shared in full. Applications for anonymised data can be made to the authors for consideration.
