# Correction to “Understanding Barriers to Engagement With a Prostate Cancer Research and Genetic Risk Service Among UK Men of Black African or Black Caribbean Ancestry”

**DOI:** 10.1111/hex.70341

**Published:** 2025-07-10

Rose, H., Hainsworth, E., Thompson, J., Green, S., Eva, M., Denzil, J., Rosalind, E. and Bancroft, E. (2025), Understanding Barriers to Engagement With a Prostate Cancer Research and Genetic Risk Service Among UK Men of Black African or Black Caribbean Ancestry. Health Expectations, 28: e70282. https://doi.org/10.1111/hex.70282

R. Hall, E. Hainsworth, J. Thompson, S. Green, E. McGrowder, D. James, R. Eeles, E. Bancroft.

**Table:** 

**Author order number**	**First name**	**Surname**
1	Rose	Hall
2	Emma	Hainsworth
3	Jeff	Thompson
4	Saran	Green
5	Eva	McGrowder
6	Denzil	James
7	Rosalind	Eeles
8	Elizabeth	Bancroft

We apologize for this error.

