# Supplementary material for: Understanding Barriers to Engagement With a Prostate Cancer Research and Genetic Risk Service Among UK Men of Black African or Black Caribbean Ancestry
Source: Health Expect. 2025 Apr 29;28(3):e70282. doi: 10.1111/hex.70282 (PMC12040735; doi:10.1111/hex.70282)
Supplement: Supplementary file 1 — Additional file 1 Topic Guide. [file HEX-28-e70282-s001.pdf]

Topic guide Structure of information and questions

Introduction
1 in 4 Black men will get prostate cancer in their lifetime. Black men are more likely to get prostate cancer than other men, who have a 1 in 8 chance of getting prostate cancer. We don't know why, but it might be linked to **genes.** We know that prostate cancer runs in some families.
We work at a centre where they do research to find out why Black men are at higher risk of prostate cancer than other men, this includes looking at **genetics.**

We want to talk to you because if we can encourage black men to take part in our research, we will be able to find out more about **why** they have a higher risk for prostate cancer. We can screen better and catch it early and treat it. There are no right or wrong answers to any of our questions, we want to hear your thoughts, whatever they are.

What type of things stop black men wanting to be part of research?

We need to know what might put black men off taking part in research about prostate cancer genetics. What do you think are the reasons? (open question – then probe with following suggestions)

- Lack of trust in doctors, nurses and hospitals
- Feeling as though you don't know enough about prostate cancer
- Feeling as though you don't know enough about genetics
- Fear of tests (needles, examinations)
- Embarrassment
- Fear of being given bad news

What kind of messages might help black men overcome any of these barriers and consider taking part in a study? (open question)

Discussion about genetics

'What, if anything, do you know about the term genetics?

How do you know this?

What does it mean to you?'

'How much do you want to know?'

'Has anyone heard of the BRCA gene?'

Prostate cancer more generally

'What sort of things would stop you acting on any symptoms you might have?'

'What would help you make a decision about getting a test?'
